# Preliminary results of targeted prostate‐specific membrane antigen imaging in evaluating the efficacy of a novel hormone agent in metastatic castration‐resistant prostate cancer

**DOI:** 10.1002/cam4.2964

**Published:** 2020-03-12

**Authors:** Chang Liu, Yao Zhu, Hengchuan Su, Xiaoping Xu, Yingjian Zhang, Shaoli Song, Beihe Wang, Dingwei Ye, Silong Hu

**Affiliations:** ^1^ Department of Nuclear Medicine Fudan University Shanghai Cancer Center Shanghai China; ^2^ Department of Urology Fudan University Shanghai Cancer Center Shanghai China

**Keywords:** abiraterone, mCRPC, PSMA, response assessment, SPECT/CT

## Abstract

To investigate the feasibility and effectiveness of prostate‐specific membrane antigen (PSMA) imaging to make response assessment regarding novel hormone treatment and to predict the outcomes for metastatic castration‐resistant prostate cancer (mCRPC) patients. This retrospective study enrolled 68 mCRPC patients who had daily received a novel hormone agent named abiraterone. Tc‐99m PSMA single‐photon emission computed tomography (SPECT/CT) was performed at the baseline (SPECT/CT1) and after 3‐6 months of treatment (SPECT/CT2). The treatment response was determined by visual analysis based on molecular imaging PSMA (miPSMA) scores framework and was compared with conventional biochemical analysis. We chose either the hottest lesion (target A) or five of the hottest lesions (target B) to calculate the tumor/background ratio (TBR) and the maximum standardized uptake value (SUVmax) and compared their performances in predicting progression‐free survival (PFS). Changes in PSMA expression between SPECT/CT1 and SPECT/CT2 were well associated with the results of the visual analysis. The TBR and the SUVmax of both targets were significantly associated with the baseline serum PSA level (*P* < .0001). The biochemical and radiological responses were concordant in 56 of the 68 patients (*P* < .001). The median PFS of the nonresponse group patients was significantly shorter than that of the patients in the response group (6.8 vs 12.1 months, *P* = .012). For predicting PFS, most of the indexes tested were significant on SPECT/CT2, with %ΔTBR being the most significant prognostic factor. Our preliminary results suggest that molecular imaging‐targeted PSMA is of great value for treatment response assessment and clinical outcome prediction in mCRPC patients with long‐term abiraterone treatment.

## Graphical abstract

We found it very accurate to evaluate the effect of abiraterone on prostate cancer using PSMA molecular imaging. PSMA imaging is expected to replace traditional imaging in prostate diagnosis and treatment

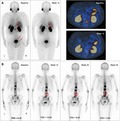

## 1 INTRODUCTION

Metastatic castration‐resistant prostate cancer (mCRPC) is an ultimately progress stage of prostate cancer (PC) that is associated with short survival and a poor prognosis.^1^ Androgen deprivation therapy (ADT) prevents PC cell proliferation by cutting off androgen receptor (AR) signaling. The novel hormonal agent abiraterone acetate, which was recently approved, has shown improvements in progression‐free survival (PFS) and overall survival (OS) in both chemotherapy‐naive and chemotherapy‐treated mCRPC patients.^2^, ^3^ However, the lack of reliable response criteria is an important limitation for clinicians because many available treatment options are not directly comparable, which creates problems in identifying the ideal sequence in which to administer treatment. Although the biochemical indicator of prostate‐specific antigen (PSA) and conventional imaging are usually used to assess the response to therapy, their results are often inconclusive.^4^, ^5^

In recent years, prostate‐specific membrane antigen (PSMA) imaging has emerged as a more sensitive and efficacious method in PC detection.^6^, ^7^ However, few studies have reported the value of PSMA imaging for evaluating the response to PC treatment.^8^, ^9^ The positron‐emission tomography (PET) response criteria in solid tumors (PERCIST) 1.0 criteria, which these studies adapted, were initially proposed for F‐18 fluorodeoxyglucose (FDG) PET.^10^ Whether this standard is feasible in PSMA imaging remains unclear. In addition, the PERCIST criteria recommend measuring the maximum standardized uptake value (SUVmax) of the hottest lesion, whereas the response evaluation criteria in the solid tumor (RECIST) 1.1 system recommend measuring the tumor diameters of a maximum of five of the hottest lesions.^11^ These differing recommendations and methods for evaluating treatment response need to be further validated for PSMA imaging.

Therefore, by combining visual molecular imaging PSMA (miPSMA) scores framework^12^ and quantitative indexes on Tc‐99m PSMA single‐photon emission computed tomography (SPECT), we aimed to investigate the feasibility and effectiveness of using PSMA imaging to evaluate the outcome of long‐term abiraterone treatment in mCRPC patients.

## 2 MATERIALS AND METHODS

### 2.1 Patient selection

This retrospective study analyzed the clinical data of all patients who were treated with abiraterone from January 2015 to December 2017 at the Fudan University Shanghai Cancer Center (FUSCC). The inclusion criteria were as follows: (a) proven mCRPC, according to the EAU guidelines^13^; (b) Tc‐99m PSMA SPECT/CT performed at both the baseline (SPECT/CT1) and after 3‐6 months (SPECT/CT2) of treatment with abiraterone, during which the PSA value was evaluated monthly; and (c) at least one PSMA‐positive lesion on SPECT/CT1. The exclusion criteria were as follows: (a) previous chemotherapy completed less than 4 weeks before SPECT/CT1 and (b) SPECT/CT1 performed more than 2 weeks before starting abiraterone treatment. The Ethics Committee of the FUSCC approved this retrospective analysis.

### 2.2 Treatment and follow‐up

Abiraterone treatment began with a standard dose and schedule: 1000 mg/day with 5 mg of prednisone twice/day continuously until it was either no longer clinically benefiting (NLCB)^4^ or until a change in treatment occurred. PFS was defined as the time from the baseline to the first of either progression of or death from PC.^14^

### 2.3 Radiopharmaceuticals and imaging protocols

The small‐molecular inhibitor of PSMA was radiolabeled using Tc‐99m, as described previously.^15^, ^16^ The patients underwent the Tc‐99m PSMA SPECT/CTs using a rotating, large field‐of‐view gamma camera (Discovery NM/CT 670, General Electric Medical Systems, Waukesha, WI) 2 hours after tracer injection. For the whole‐body planar image, the following parameters were chosen: the main energy window was 140 keV ± 10%; the scatter energy window was 120 keV ± 5%; the matrix size was 256 × 1,024; and the scan speed was 15 cm/min.

### 2.4 Image analysis

The image reconstruction was performed on a workstation (Xeleris, General Electric, Waukesha, WI). All scans were reviewed and interpreted by two experienced nuclear‐medicine specialists who were blinded to the clinical data. Lesions were classified into four organ systems: prostate/prostate bed, lymph nodes, bone, and visceral metastatic sites. Any visually determined lesions were scored using the miPSMA framework, which includes a 4‐point scale that was proposed by Eiber et al in 2018.^12^ In addition to visual analysis, quantitative SPECT/CT analysis was carried out for all patients. The tumor/background ratio (TBR) was calculated using the quotient of maximal counts within circular volumes of interests (VOIs) and the mean counts within the obturator muscle.^17^ The counts in the images were converted into kilobecquerels per milliliter in units of kBq/ml, as shown in a previous study, using a calibration factor that was derived from a phantom experiment^18^ and then normalized by patient weight and the injected dose to yield the respective SUVmax.

Two sets of target lesions were defined: the hottest lesion (target A) and a maximum of 5 of the hottest lesions, no more than 2 of which were in a single organ (target B). In target B, the quantitative SPECT indexes of all lesions were summed into a single value. At SPECT/CT1 and SPECT/CT2, the quantitative indexes were measured and their percent differences (%Δ) were calculated.

### 2.5 Therapy response assessment

The responses to abiraterone were assessed biochemically and radiologically. The PSA response was defined as a decrease in the PSA level of ≥ 50% from the baseline value.^19^ Any increase or decrease in absolute PSA values between the PSA1 (baseline) and PSA2 (SPECT/CT2) was considered a trend toward an increasing or decreasing PSA, respectively. A PSA flare was defined as a PSA that initially increased under abiraterone therapy and thereafter dropped to values below those at the baseline.^20^

The radiological response was determined according to the miPSMA scores of SPECT/CT. There was deemed to be no radiological response if miPSMA scores either increased or persisted within 3 points during treatment or if new foci emerged that were compatible with metastasis. A radiological response was defined as the presence of either decreased miPSMA scores or a score that persisted within 1‐2 points during treatment with no new lesions. New PSMA uptake that was seen on SPECT2 was confirmed as PC lesions using either clinical follow‐up or rebiopsy. Additional follow‐up information included monthly PSA levels, contrast‐enhanced CTs, magnetic resonance imaging (MRIs), and bone scans. A PSMA flare phenomenon was defined as a new PSMA uptake together with a PSA level that had decreased ≥ 50% and with no evidence of disease progression on the next 3‐ to 6‐month imaging scan.

### 2.6 Statistical analysis

Analysis of variance (ANOVA) and t tests were used for continuous variables, and chi‐squared tests were used for categorical variables. We used McNemar's test to compare the concordances between radiographic and biochemical responses. The optimal cutoff values for SPECT/CT parameters were determined by time‐dependent survival receiver operating characteristic (ROC) analysis (survival ROC library in R), which took into account the duration of time until censoring or progression.^21^ The optimal cutoff points were used to discriminate high‐ and low‐value groups, as well as for plotting. To identify independent prognostic factors for outcome, hazard ratios (HRs) and their 95% confidence intervals (CIs) were determined for each variable using the Cox univariate model of regression. The PFS was also analyzed using the Kaplan‐Meier curve, and the log‐rank test to assess any differences between outcome curves. The Statistical Package for the Social Sciences (SPSS) Version 19.0 software (SPSS Inc) was used to conduct statistical analyses. *P* values less than .05 were considered statistically significant.

## 3 RESULTS

### 3.1 Patient characteristics

This retrospective study enrolled 68 eligible mCRPC patients. At SPECT/CT1, 58 patients (85%) showed evidence of bone metastases. Visceral metastases were found in nine patients (13.2%) with bone or lymph‐node metastases, and three patients (4%) showed local recurrence. The baseline average PSA level was 68.2 ng/mL, and the average abiraterone treatment time was 179 days. The clinical characteristics are summarized in Table 1.

**Table 1:** Patient demographics and clinical characteristics

Demographic or clinical characteristic	value
No. of patients	68
Age (years)	70 (median; range 61‐84)
Tracer (MBq)	734 (median; range 643‐798)
PSA before abiraterone treatment (ng/mL)	19.2 (median; range 1.76‐918.03)
Gleason score	
<8	10 (14.7%)
≥8	58 (85.3%)
Primary therapies	
Radical prostatectomy	42 (61.8%)
Radical prostatectomy + traditional ADT	10 (14.7%)
External beam radiotherapy	3 (4.4%)
External beam radiotherapy + traditional ADT	11 (16.2%)
Traditional ADT only	2 (2.9%)
Chemotherapy before AA treatment	
Yes	21 (30.9%)
No	47 (69.1%)
AA treatment time (day)	157 (median; range 95‐377)
Follow‐up time (month)	18.3 (median; range 6.7‐34.8)
Sites of disease	
Local recurrence + other metastases	3 (4.4%)
Lymph node only	10 (14.7%)
Bone only	25 (26.5%)
Lymph node + bone	29 (42.6%)
Lymph node + visceral organs	5 (7.3%)
Bone + visceral organs	2 (2.9%)
Lymph node + bone +visceral organs	2 (2.9%)

### 3.2 Comparative analysis of biochemical and radiological results

In visual analysis of SPECT/CT2, 36 patients were classified as the response group, and the remaining 32 patients were classified as the nonresponse group, according to the criteria mentioned above. A PSA response was seen in 28 patients (41.2%). Overall, biochemical and radiological responses were concordant in 56 of the 68 patients (κ = 0.77; 95% CI, 2.44‐6.84; *P* < .001). A trend toward increasing PSA was seen in 27 patients (39.7%), but 6 of them were evaluated as responding at PSMA SPECT/CT2. A trend toward decreasing PSA was seen in 41 patients (60.3%), but 11 of them were evaluated as nonresponders at PSMA SPECT/CT2. Two of the 11 patients achieved a PSA response (Table 2).

**Table 2:** Comparison between radiological and biochemical response to abiraterone treatment

PSA status	Patients number	PSMA response	PSMA no response
Increasing PSA trend	27	6 (22.2%)	21 (77.8%)
Decreasing PSA trend	41	30 (73.2%)	11 (26.8%)
PSA decrease ≥ 50%	28	26 (92.9)	2 (7.1%)
0%< PSA decrease < 50%	13	4 (30.8%)	9 (69.2%)

**Figure 1:**
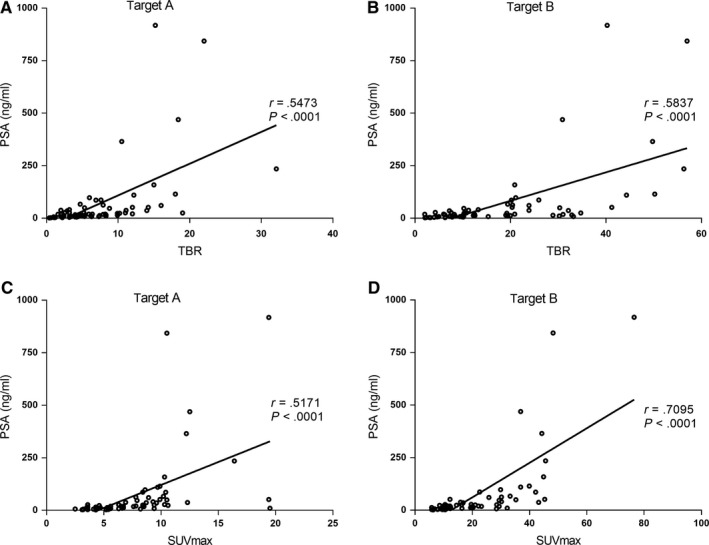
Correlations between TBR (A, B) and SUVmax (C, D) of both targets and the serum PSA level

At the baseline, the TBR (Figure 1A, B) and SUVmax (Figure 1C, D) values of both targets were significantly positively related to the PSA level (*P* < .0001). The %ΔSUVmax and %ΔTBR between SPECT/CT1 and SPECT/CT2 were well associated with the visual analysis and were significantly different between the response and nonresponse groups for both targets A and B (Table 3).

**Table 3:** Baseline and follow‐up values of quantitative indexes according to response by visual analysis

Index	SPECT/CT1	SPECT/CT2			%Δ between SPECT/CT1 and SPECT/CT2		
		Response	Nonresponse	*P*	Response	Nonresponse	*P*
Target A							
TBR	7.3 ± 5.8	4.9 ± 4.1	6.8 ± 1.9	.1923	66.2 ± 7.9	28.9 ± 123.4	.0023*
SUVmax	7.5 ± 3.8	4.3 ± 2.7	8.8 ± 3.5	.0542	78.2 ± 10.2	32.2 ± 157.8	.0083*
Target B							
TBR	17.8 ± 13.8	10.2 ± 5.7	16.9 ± 6.8	.0624	61.9 ± 33.2	18.5 ± 179.3	<.001*
SUVmax	20.8 ± 13.9	12.2 ± 3.9	25.8 ± 4.4	.0787	69.5 ± 12.9	20.2 ± 111.6	<.001*

*P < .05.

### 3.3 Tumor flare phenomenon

**Figure 2:**
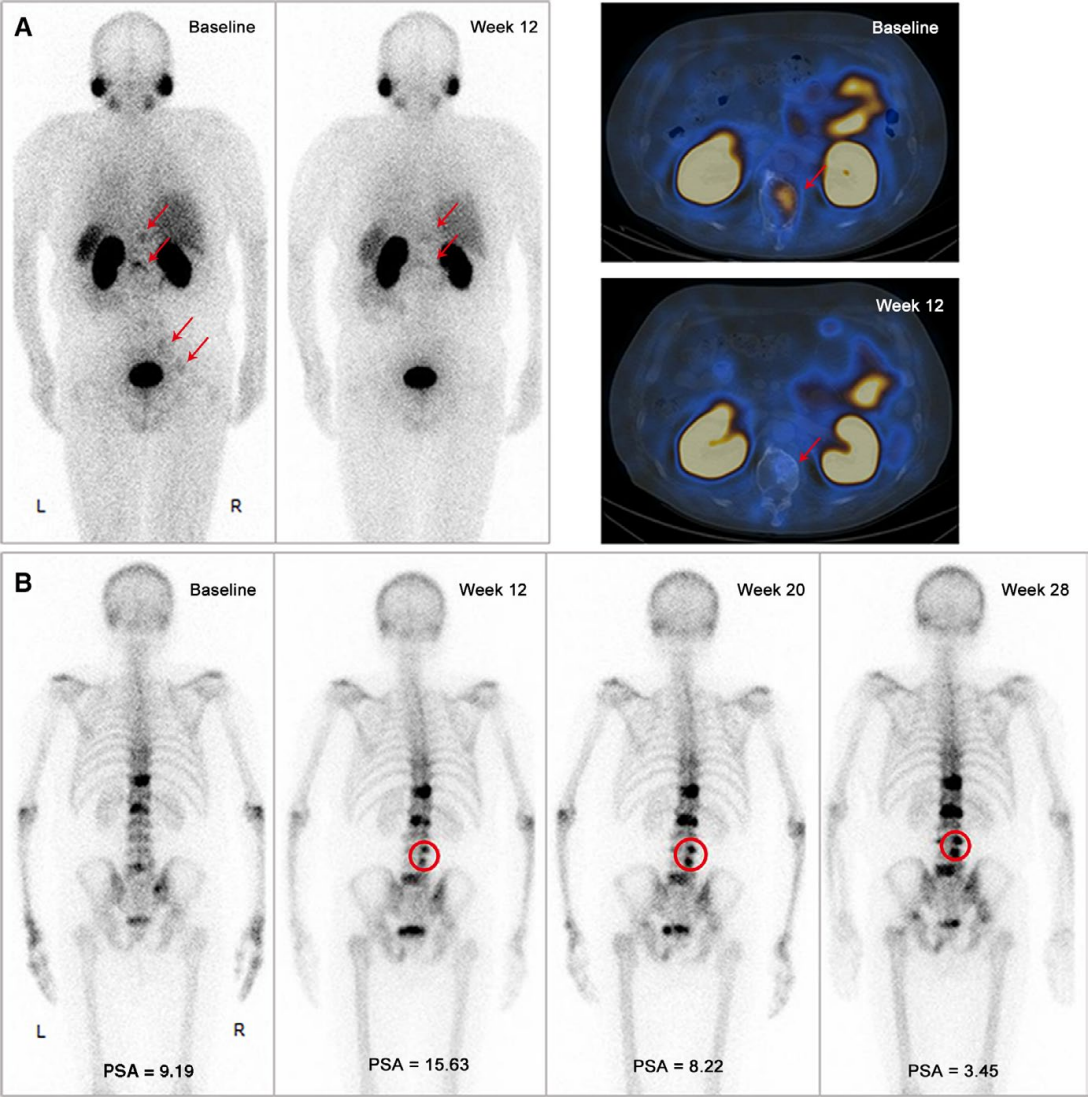
A 70‐yr‐old mCRPC patient with Gleason 4 + 5 who progressed after 20 months on traditional hormone treatment, and 8 docetaxel cycles of chemotherapy. PSMA imaging both before and after 12 weeks of abiraterone treatment showed a decrease in the miPSMA score of the bone lesions (A, red arrow). However, at week 12, there was a significant increase in the PSA and 2 new lesions on the bone scan (B, red circle). By weeks 20‐28, the PSA progression improved, and no additional lesions appeared on the bone scan, indicating that the progression seen at week 12 was due to PSA and a bone flare

Of the 27 patients who had a trend toward increasing PSA, 6 (8.8%) showed a PSA flare. They all were classified as part of the response group at SPECT/CT2. No suspected PSMA SPECT/CT flare phenomena occurred. Additional bone scan follow‐ups were assessed in 12 patients, of which 2 showed a bone flare phenomenon according to the “2 + 2” principle in the Prostate Cancer Working Group 3 (PCWG3) criteria.^4^ PSMA SPECT/CT evaluation was also more accurate in these two patients. Only 1 patient had both PSA and bone scan flare (Figure 2B), who showed decreased miPSMA scores on SPECT/CT2 (Figure 2A). After 17 months of follow‐up, he was continuously progression free.

### 3.4 Predictive value of treatment outcomes

After a median follow‐up of 18.3 months, 83.8% of the patients had documented disease progression (n = 57) and only 11.8% had died (n = 8). The data on OS were unreliable due to the low number of deaths. The median PFS was 8.4 months (95% CI: 5.5‐8.1).

**Figure 3:**
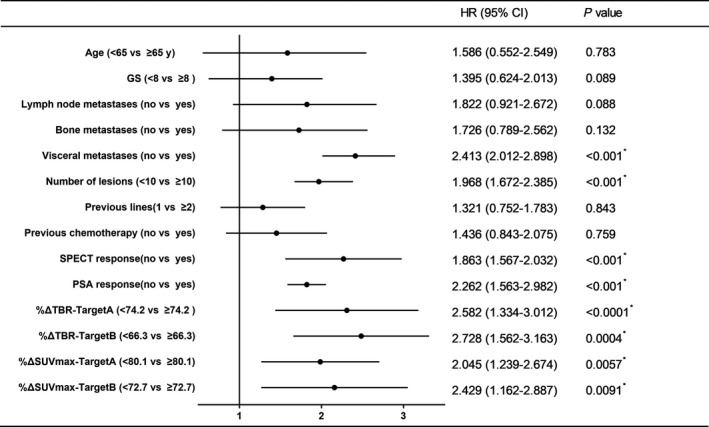
Prognostic values of clinical risk factors and quantitative SPECT indexes in predicting progression‐free survival

Regarding the conventional clinical risk factors, the results showed that shorter PFS was significantly associated with the number of lesions being > 10, visceral metastases, and the lack of a PSA response (*P* < .001) (Figure 3).

**Figure 4:**
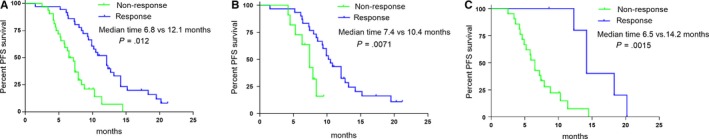
Kaplan‐Meier survival curves for PFS, according to a visual analysis. A, All patients; B, patients with an increasing PSA trend; C, patients with a decreasing PSA trend

**Figure 5:**
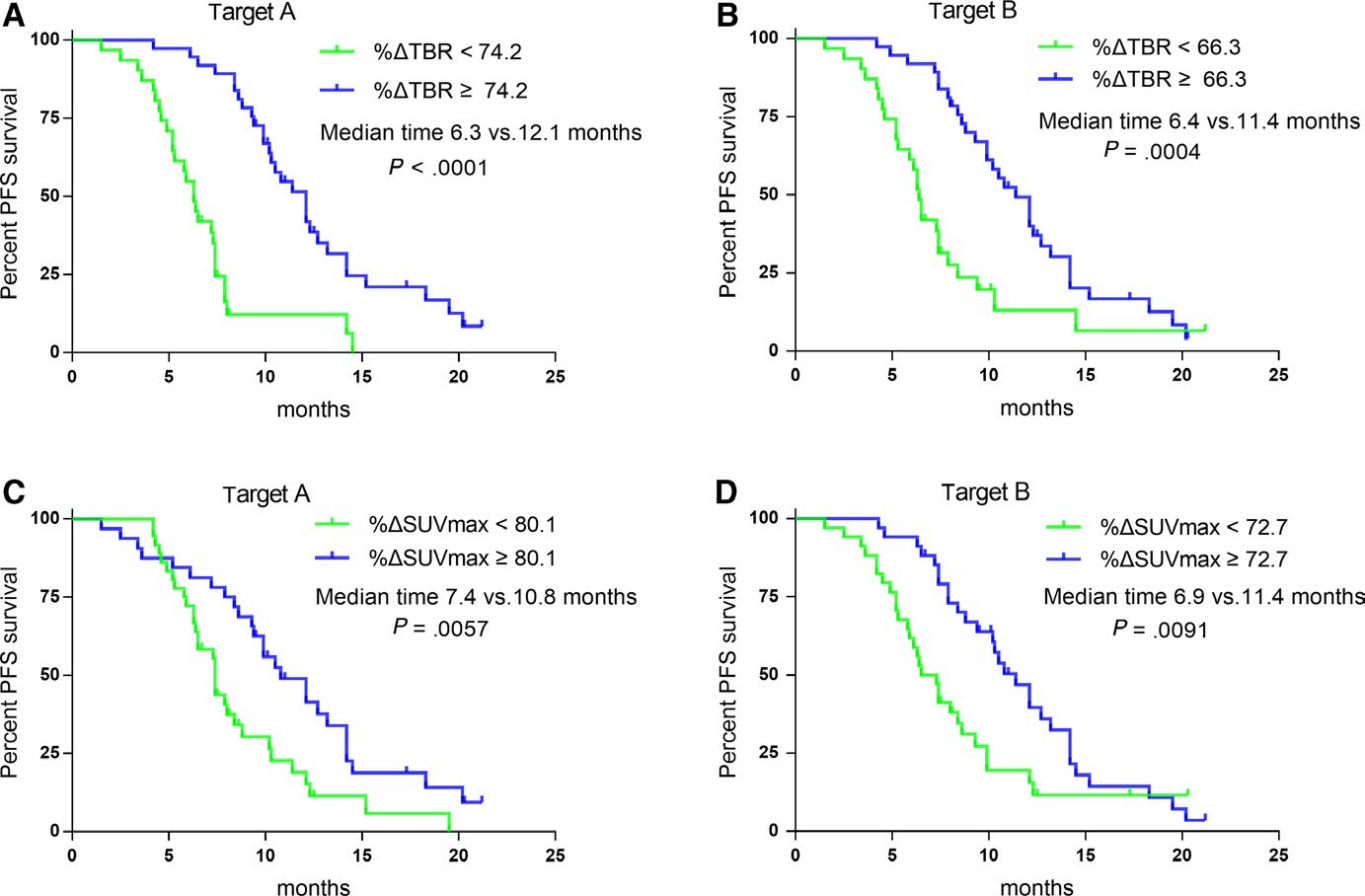
Kaplan‐Meier survival curves for PFS, according to %ΔTBR and %ΔSUVmax, for target A (A, C) and target B (B, D)

In the visual analysis, the median PFS of patients in the nonresponse group at SPECT/CT2 was 6.8 months, which was significantly shorter than the 12.1 months for patients in the response group (*P* = .012) (Figure 4A). Moreover a subgroup analysis of patients who had trends toward decreasing or increasing PSAs found a significant difference in PFS between patients who were nonresponders and those who responded at SPECT/CT2 (*P* = .0071 and .0015, respectively) (Figure 4B, C). At SPECT/CT2, both %ΔTBR and %ΔSUVmax were significant prognostic factors (Figure 3), whereas SUVmax and TBR were not. Univariate analyses were then performed, and the optimal cutoff value for PFS was determined using time‐dependent ROC analysis. In target A, the median PFS of patients with high %ΔTBR (>74.2) was 12.1 months, significantly longer than 6.3 months in patients with low %ΔTBR (<74.2, *P* < .0001; Figure 5A). The median PFS of patients with high %ΔSUVmax (>80.1) was also significantly longer than patients with low %ΔSUVmax (<80.1; 10.8 vs 7.4 months, *P* = .0057; Figure 5C). In terms of target B, high %ΔTBR (>66.3) and %ΔSUVmax (>72.7) were also significant for better outcomes (Figure 5B/D). Among them, %ΔTBR presented the highest HR in targets A and B (2.58 and 2.73, respectively).

## 4 DISCUSSION

Compared with conventional imaging, molecular imaging has unique advantages in assessing treatment response, and clinical studies have demonstrated that F‐18 FDG PET/CT can predict the response in cases with several tumors.^10^, ^22^ However, it is still unclear whether a receptor‐targeting radiopharmaceutical would be as valuable as a metabolic tracer for monitoring treatment response in PCa. Seitz et al^8^ firstly focused on using Ga‐68 PSMA PET/CT to assess therapy response in metastatic PC patients’ treatment with docetaxel chemotherapy. Their conclusion was that Ga‐68 PSMA PET/CT might be superior to conventional CT for assessing therapy response. Next, Schmidkonz et al evaluated the usefulness of Tc‐99m MIP‐1404 SPECT/CT for assessing early response in 3 small cohorts of metastatic PC patients.^9^ In the ADT treatment group, the agreement rate between SPECT/CT and PSA was 75%, which was higher than that of CT. However, the sample sizes in these studies were small, and the heterogeneity between the patients was high.

To the best of our knowledge, there are few studies on response evaluation with a follow‐up PSMA SPECT/CT after hormone treatment in patients with mCRPC. We not only studied the relationship between PSMA SPECT/CT response and conventional evaluation but also demonstrated the rationality of PSMA evaluation through survival data. Notably, in determining whether PSMA imaging can be used to evaluate the efficacy of hormone treatment, the most important point is to clarify the relationship between PSMA expression and hormone treatment. We know from previous studies that luteinizing hormone releases hormone analogues and AR blockers, including bicalutamide and enzalutamide, which can obviously increase PSMA expression in PC cells, animal models, and small samples of patients.^23^, ^24^ However, in these studies, the duration of hormone treatment was usually less than 30 days. Afshar‐Oromieh et al demonstrated that the majority of PC lesions were no longer visible in patients who were receiving an average of 229 days of effective hormone treatment.^25^ Lückerath et al showed that enzalutamide‐induced PSMA expression neither retards tumor growth nor prolongs survival more than does Lu‐177 PSMA‐617 treatment alone.^26^ Schlenkhoff et al found that a 71‐year‐old mCRPC patient showed a very good response on Ga‐68 PSMA‐11 PET/CT after 4 months of hormone treatment.^27^ These studies suggested that the upregulation of PSMA expression by hormone treatment was transient and nonqualitative. The optimal timing of the PSMA imaging that was used to assess therapy response was essential to reducing the false‐positive uptake and improving the specificity. In this study, we chose 3‐6 months as the cutoff evaluation time‐point for retrospective analysis and obtained encouraging results. We confirmed that PSMA SPECT/CT could be a marker for novel hormone treatment response in mCRPC. Using visual analysis based on miPSMA scores, clinicians can make judgments both easily and accurately. Moreover, the median PFS of the nonresponse group was significantly shorter than that of the response group (6.8 vs 12.1 months). This suggested that the increased expression of PSMA after long‐term hormone treatment represented disease progression rather than AR inhibition. Furthermore, this study found that no flare phenomena occurred on PSMA post‐treatment imaging. This indicated that, unlike the bone‐scan “2 + 2” criterion, there was a high incidence of metastases in new lesions that were highly PSMA expressed.

The measurement of serum PSA is the most important pertinent biological marker for men, leading many physicians to watch for PSA changes to assess the efficacy of treatment in patients with mCRPC. In previous studies, a PSA decline of ≥50% that was confirmed ≥4 weeks after the initiation of treatment was consensually considered the gold standard for an effective response.^8^, ^9^ However, in this series, a mismatch between the Tc‐99m PSMA SPECT/CT and the PSA response was seen in 12 of the 68 patients. The reasons for this were manifold. For example, a PSA flare phenomenon during abiraterone treatment was observed in 6.0%‐10.8% of the mCRPC patients, and it could appear as much as 3 months after the initiation of treatment.^28^ Although the appearance of this phenomenon did not affect either PFS or OS,^29^ it could have negatively affected clinical decisions and thus have to early withdrawal. Therefore, in our center, the patients were treated with abiraterone until NLCB, according to the PCWG3 criteria.^4^ This resulted in our identifying the PSA flare phenomenon during the evaluation time (in six patients), and these patients were all classified in the response group by their PSMA SPECT/CT2 results. In addition, it remains uncertain whether a decline in the PSA level can be used as a true surrogate for predicting survival in patients with mCRPC. For example, radium‐223 was recommended to treat bone metastasis from CRPC.^19^ It did not decrease PSA levels, but it did prolong OS compared with a placebo,^30^ raising questions about PSA levels as an estimator of OS. Meanwhile, the selection pressure and lineage plasticity of AR‐pathway inhibition can lead to neuroendocrine differentiation of CRPC, as is the case with bulky metastatic disease that has low serum PSA levels.^31^ Elevated neuron‐specific enolase (NSE) and circulating chromogranin A (CgA) have been reported as possible biological markers in which the role of PSMA imaging remains unclear. In our subgroup analysis of patients who had a trend toward decreasing PSA, there was a significant difference in PFS between patients who did not respond and those who responded at SPECT/CT2. This suggested that PSMA SPECT/CT may be able to identify disease progression earlier than PSA levels can. Therefore, further prospective research is needed on whether the integrated use of PSMA imaging and other biological markers could potentially significantly improve outcome prediction for mCRPC patients.

Our preliminary study has several limitations. Firstly, most patients had already received multiple different therapies, which may have influenced the PFS. However, this is inevitable for advanced mCRPC patients. In addition, the miPSMA score criteria that were initially used to evaluate the classification of PSMA expression had never been adapted as a criterion for evaluating efficacy in the past. A concern that the existing image evaluation criteria are not applicable to PSMA imaging, and some phenomena, including halo artifacts, may also affect the accuracy of the SUV values.^32^ This indicates the need to look for new standardized PSMA criteria, and this study was a preliminary exploration in this regard. Finally, the study's retrospective design could be considered another limitation. Larger, multicenter prospective clinical trials are needed.

## 5 CONCLUSION

This study found that miPSMA score‐based visual analysis is suitable for treatment response assessment. The use of molecular imaging–targeted PSMA is of great value for both treatment response assessment and clinical outcome prediction in mCRPC patients with long‐term abiraterone.

## CONFLICTS OF INTEREST

None.

## AUTHOR CONTRIBUTIONS

Study design/methodology: Chang Liu, Silong Hu, Dingwei Ye, Yao Zhu, Yingjian Zhang, and Shaoli Song. Data acquisition: Hengchuan Su and Beihe Wang. Data analysis: Chang Liu, Beihe Wang, and Hengchuan Su. Radiopharmaceuticals preparation: Xiaoping Xu. Writing manuscript: All coauthors.

## ETHICS APPROVAL NUMBER

The Ethics Committee of FUSCC approved this retrospective analysis (1506148‐4‐1612A).

## Data Availability

The data used and/or analyzed for this study are available from the corresponding author at reasonable request.
